# Supplementary material for: Role and mechanism of NCAPD3 in promoting malignant behaviors in gastric cancer
Source: Front Pharmacol. 2024 Apr 22;15:1341039. doi: 10.3389/fphar.2024.1341039 (PMC11070777; doi:10.3389/fphar.2024.1341039)
Supplement: Supplementary file 11 [file DataSheet2.ZIP › GSEA/Transcipt factor/my_analysis.Gsea.1599461814734/HFH4_01.html]

Details for gene set HFH4\_01[GSEA]

|  || Dataset | filtered\_dataset.sample\_info.cls#WT\_versus\_NCAPD3\_MUT |
| Phenotype | sample\_info.cls#WT\_versus\_NCAPD3\_MUT |
| Upregulated in class | NCAPD3\_MUT |
| GeneSet | HFH4\_01 |
| Enrichment Score (ES) | -0.4492883 |
| Normalized Enrichment Score (NES) | -2.0177393 |
| Nominal p-value | 0.005221932 |
| FDR q-value | 0.06473612 |
| FWER p-Value | 0.531 |
Table: GSEA Results Summary

  

Fig 1: Enrichment plot: HFH4\_01      
 Profile of the Running ES Score & Positions of GeneSet Members on the Rank Ordered List

  

| SYMBOL | TITLE | RANK IN GENE LIST | RANK METRIC SCORE | RUNNING ES | CORE ENRICHMENT || 1 | 9315 | NREP | 364 | 0.557 | -0.2143 | No |
| 2 | 79047 | KCTD15 | 469 | 0.493 | -0.2473 | No |
| 3 | 146691 | TOM1L2 | 472 | 0.490 | -0.2072 | No |
| 4 | 5567 | PRKACB | 595 | 0.426 | -0.2588 | No |
| 5 | 9223 | MAGI1 | 737 | 0.357 | -0.3298 | No |
| 6 | 31 | ACACA | 780 | 0.334 | -0.3318 | No |
| 7 | 23710 | GABARAPL1 | 913 | -0.341 | -0.3977 | No |
| 8 | 9572 | NR1D1 | 977 | -0.392 | -0.4098 | Yes |
| 9 | 9586 | CREB5 | 1009 | -0.409 | -0.3974 | Yes |
| 10 | 80315 | CPEB4 | 1053 | -0.448 | -0.3904 | Yes |
| 11 | 604 | BCL6 | 1136 | -0.504 | -0.4066 | Yes |
| 12 | 639 | PRDM1 | 1159 | -0.523 | -0.3781 | Yes |
| 13 | 4651 | MYO10 | 1213 | -0.588 | -0.3663 | Yes |
| 14 | 861 | RUNX1 | 1293 | -0.687 | -0.3649 | Yes |
| 15 | 3084 | NRG1 | 1320 | -0.739 | -0.3210 | Yes |
| 16 | 57157 | PHTF2 | 1334 | -0.755 | -0.2664 | Yes |
| 17 | 5156 | PDGFRA | 1383 | -0.932 | -0.2220 | Yes |
| 18 | 51274 | KLF3 | 1406 | -1.296 | -0.1281 | Yes |
| 19 | 255394 | TCP11L2 | 1408 | -1.538 | 0.0014 | Yes |
Table: GSEA details [plain text format]

  

Fig 2: HFH4\_01      
 Blue-Pink O' Gram in the Space of the Analyzed GeneSet

  

Fig 3: HFH4\_01: Random ES distribution      
 Gene set null distribution of ES for **HFH4\_01**
